# Effect of Gluten-Free Diet on Metabolic Control and Growth Parameters Among Children and Adolescents With Type 1 Diabetes During the First Year After Diagnosis of Celiac Disease: A Retrospective Case–Control Study

**DOI:** 10.1155/pedi/1283259

**Published:** 2025-07-04

**Authors:** Ahmed Monir Hegab, Ashraf Abou-Taleb

**Affiliations:** Pediatrics Department, Faculty of Medicine, Sohag University, Sohag, Egypt

**Keywords:** celiac disease, children and adolescents, glycemic control, growth parameters, type 1 diabetes

## Abstract

**Aims:** Assessment of celiac disease and gluten-free diet (GFD) associations with metabolic control and growth parameters in children and adolescents with type 1 diabetes mellitus (T1DM) during the first year after diagnosis of celiac disease.

**Methods:** This was a retrospective case–control study that included 47 children and adolescents with T1DM aged <18 years who attended the pediatric diabetes clinic at Sohag University Hospital, Egypt, and had a biopsy-proven diagnosis of celiac disease between January 2017 and December 2021. Each case had two age-, sex-, and duration of diabetes-matched control participants with T1DM who had persistently negative celiac screening tests. Clinical characteristics, growth parameters, insulin doses, celiac autoantibody titers, and HbA1c levels throughout the first year after diagnosis of celiac disease were obtained from the medical records.

**Results:** Children and adolescents with celiac disease had significantly lower insulin doses at diagnosis (*p*=0.002) compared to their matched controls. There were no significant differences between both groups regarding the HbA1c levels at diagnosis of celiac disease or after 1 year (*p*=0.27 and 0.81, respectively). Patients with celiac disease had significantly lower weight, height, and body mass index (BMI) standard deviation scores (SDSs) at diagnosis and after 1 year. There were no significant differences between both groups regarding the fasting lipid profiles at diagnosis or after 1 year. Patients with villous atrophy at diagnosis had significantly higher HbA1c levels after 1 year (*p*=0.04). There were no significant improvements in weight, height, and BMI SDS after 1 year even in patients with normalized celiac autoantibodies.

**Conclusions:** Children and adolescents with T1DM had lower insulin requirements and growth parameters at diagnosis of celiac disease. Villous atrophy at diagnostic small bowel biopsies was associated with worsening glycemic control after 1 year. Longer follow-up periods are required to detect significant improvements in growth parameters.

## 1. Introduction

Type 1 diabetes mellitus (T1DM) is an autoimmune disease characterized by a progressive destruction of pancreatic beta cells [1]. Other autoimmune diseases, such as Hashimoto's thyroiditis, vitiligo, Addison's disease, and celiac disease, are commonly associated with T1DM due to the common genetic background of these disorders [2]. Children and adolescents with T1DM should have a scheduled screening for these diseases as a part of their follow-up programmes [3].

Celiac disease is a common autoimmune disease that affects 1%–2% of the general population [4]. The prevalence of celiac disease is higher among children and adolescents with T1DM [5, 6]. Most cases of celiac disease are detected in asymptomatic or mildly symptomatic children and adolescents with T1DM during routine screening for celiac autoantibodies [7, 8].

Children and adolescents with celiac disease should follow a strict lifelong gluten-free diet (GFD), avoiding all foods containing wheat, rye, and barley [9]. Adherence to GFD imposes an additional burden on children and adolescents with T1DM and their families [6]. These children and adolescents already have to follow a diet regimen to control their T1DM. They have to count the carbohydrate contents of each meal and adjust their insulin doses according to their insulin-to-carbohydrate ratio [10, 11]. The psychological difficulties in accepting a GFD may negatively impact their glycemic control [12].

Management of celiac disease with GFD may affect glycemic control and growth parameters among children and adolescents with T1DM through different mechanisms. The healing of intestinal mucosa following adherence to GFD might increase the intestinal absorption of glucose and other nutrients. This may affect glycemic control and growth parameters among those children and adolescents [13–15]. Moreover, most gluten-free foods have higher glycemic indices compared to gluten-containing products, leading to higher postprandial blood glucose levels following gluten-free meals [6].

Several studies aimed to assess the effects of celiac disease and GFD on the glycemic control of children and adolescents with T1DM [13–18]. Earlier studies conducted on children with symptomatic celiac disease reported improvements in glycemic control on GFD [19, 20]. However, this effect was not well-established in further studies on asymptomatic children and adolescents detected by routine screening [21, 22]. Some studies found an increase in glycosylated hemoglobin A1c (HbA1c) levels or the total daily insulin doses (TDDs) among children and adolescents with T1DM after the commencement of GFD [13–17]. Other studies reported improvements in glycemic control among children and adolescents with T1DM while on GDF [23, 24]. However, only a few previous studies aimed to assess the effect of GDF on lipid profiles among children and adolescents with T1DM and celiac disease [25, 26].

Moreover, previous studies that assessed the effects of GFD on growth parameters for children and adolescents with T1DM and celiac disease reported contradictory results. Some studies demonstrated improvements in growth parameters following the use of GFD [13, 18, 27]. Other studies found no significant changes in the weight, height, or body mass index (BMI) among children and adolescents with T1DM following the diagnosis and treatment of celiac disease [15, 17, 28].

Therefore, this study aimed to assess celiac disease and GFD associations with metabolic control and growth parameters in children and adolescents with T1DM during the first year after diagnosis of celiac disease.

## 2. Patients and Methods

### 2.1. Study Setting and Inclusion Criteria

This was a retrospective case–control study. Children and adolescents with T1DM, aged <18 years, who attended the pediatric diabetes clinic at Sohag University Hospital, Egypt, and had a biopsy-proven diagnosis of celiac disease between January 2017 and December 2021 were included as cases. Children and adolescents who were diagnosed with celiac disease before the onset of T1DM were excluded.

For each case with T1DM and celiac disease, two age-, sex-, and duration of diabetes-matched children and adolescents with T1DM who had persistently negative celiac autoantibodies screening tests during the same period were included as controls.

### 2.2. Study Population

Throughout the 5-year study period, 1032 children and adolescents with T1DM were assessed at our pediatric diabetes clinic. Among them, 815 (78.9%) were screened at least once for celiac disease. A total of 374 (45.9%) of the screened children and adolescents were females. The median age at onset of T1DM for the screened children and adolescents was 7 (4–10) years and the median duration of T1DM at first celiac screening was 1.25 (1.00–1.50) years.

Among the screened children and adolescents, 71 children and adolescents (8.7%) had positive celiac autoantibodies. Small bowel biopsies were performed on 62 children and adolescents. Of them, nine had normal biopsies (Marsh stage 0). The remaining 53 children and adolescents had biopsy-proven celiac disease (Marsh stage ≥ 1). Six children and adolescents were excluded from the study because their medical records were incomplete. The remaining 47 children and adolescents were included in the study as cases. Ninety-four age-, sex-, and duration of diabetes-matched children and adolescents with T1DM who had persistently negative celiac screening tests during the same study period were included as a control group.

### 2.3. Diagnosis and Management of the Study Participants

#### 2.3.1. Diagnosis and Management of T1DM

In our pediatric diabetes unit, diagnosis of diabetes mellitus in children and adolescents was done based on the American Diabetes Association clinical, blood glucose, and glycosylated hemoglobin criteria [29]. Diagnosis of T1DM was established in insulin-dependent children and adolescents after exclusion of clinical criteria for type 2 diabetes mellitus, monogenic, secondary, and drug-induced diabetes mellitus [30, 31]. The diagnosis of T1DM was confirmed by detecting positive Islet cell autoantibodies and low fasting and post-prandial serum C-peptide levels at diagnosis and after 6 months [30, 32].

All children and adolescents with T1DM attending our pediatric diabetes clinic were treated with the multiple daily insulin injection regimen [33]. Rapid-acting insulin analogs (insulin lispro [Humalog], insulin aspart [Novorapid], or insulin glulisine [Apidra]) were used as bolus insulin for premeal doses. A long-acting insulin analog (insulin glargine 100 IU/mL [Lantus] or insulin degludec 100 IU/mL [Tresiba]) was given as a once-daily basal dose.

All children and adolescents with T1DM were trained on different methods of carbohydrate counting [10, 11] and were instructed to keep their carbohydrate intake within the recommended daily requirements [34]. They used a flexible advanced carbohydrate counting regimen with adjustments of bolus insulin doses according to the carbohydrate content of each meal or snack [10, 11].

#### 2.3.2. Celiac Disease Screening Tests and Diagnostic Criteria

In our pediatric diabetes unit, the first screening for celiac disease in asymptomatic children and adolescents with T1DM was done 6 months after diagnosis of T1DM. Screening for celiac disease was repeated annually for the first 5 years after diagnosis of T1DM, then every 2 years thereafter. Screening for celiac disease was not done at the diagnosis of diabetes mellitus for two reasons. First, to give enough time for children and adolescents with T1DM and their families to master carbohydrate counting methods before introducing the GFD. The simultaneous introduction of two dietary regimens imposes an additional burden on those children and adolescents and might impair their adherence to both regimens. Second, to give time to exclude other types of diabetes mellitus that might be mistaken as T1DM at the disease onset and avoid doing unnecessary screening for autoimmune diseases in a non-autoimmune type of diabetes mellitus [30, 31].

Children and adolescents with T1DM were screened for celiac disease using tissue transglutaminase IgA (TTG-IgA) antibodies using standard commercial enzyme-linked immunosorbent assay tests. Total serum IgA levels were also measured for all children and adolescents with T1DM using commercial kits. If the total serum level of IgA was below the lower limit of normal, as determined by the manufacturer, the TTG-IgG was assessed. The diagnosis of celiac disease was confirmed by biopsy in children and adolescents with positive TTG-IgA or TTG-IgG [35].

The biopsies were taken by upper gastrointestinal endoscopy performed by a specialized pediatric gastroenterologist at Sohag University Hospital. At least one biopsy from the duodenal bulb and four biopsies from the distal duodenum were taken. The histopathological assessment was performed by a specialized histopathologist at Sohag University Hospital on correctly oriented biopsies. The histopathological features of the biopsy specimen were classified according to Marsh [36] classification which includes 4 stages: Stage 0 (normal mucosa), Stage 1 (increased lymphocytic infiltration of the mucosa >30 cells per 100 epithelial cells), Stage 2 (crypt hyperplasia), and Stage 3 (villous atrophy whether partial [3a], subtotal [3b], or total [3c]).

#### 2.3.3. Management and Follow-up of Children and Adolescents With Celiac Disease

Children and adolescents with positive TTG-IgA or TTG-IgG and abnormalities in the mucosa (Marsh stages 1–3) were instructed to follow a strict GFD. Children and adolescents with celiac disease and their families were educated about the role of dietary gluten in celiac disease development and the importance of following a strict GFD by a specialized dietitian [37]. They were also given written information leaflets about gluten-free food and gluten-containing food and how to safely prepare gluten-free meals without gluten contamination.

Adherence to GFD was assessed by monitoring the celiac autoantibody status every 6 months. Children and adolescents with celiac disease who failed to achieve and maintain a negative celiac autoantibody status on GFD during the first year after diagnosis of celiac disease were considered to be nonadherent to GFD [38].

### 2.4. Data Collection

The medical files of the included children and adolescents were reviewed. Clinical characteristics, growth parameters (weight, height, and BMI), insulin doses, celiac autoantibody titers, HbA1c levels, and fasting serum lipid profiles at the time of diagnosis of celiac disease for the cases or at the time of the negative screening tests for the controls and after 1 year were obtained from the medical records for each patient.

The weight and height were measured by trained nurses at the pediatric diabetes clinic. Weights were measured with light clothes and no shoes using digital scales and approximated to the nearest 0.1 kg. Heights were measured bare-footed using a stadiometer and approximated to the nearest 0.5 cm. The BMI was calculated as the weight in kilograms divided by the square of the height in meters [39]. Standard deviation scores (SDSs) for weight-for-age, height-for-age, and BMI were calculated using WHO standard references [40, 41].

HbA1c levels were measured using high-performance liquid chromatography calibrated against the National Glycosylated Standardization Programme (NGSP). Fasting serum lipid profiles, including total cholesterol (TC), triglycerides (TGs), low-density lipoprotein (LDL) cholesterol, and high-density lipoprotein (HDL) cholesterol, were measured in the morning after 8 h of fasting using automatic biochemistry analyzers by an enzymatic assay.

### 2.5. Ethical Considerations

The study protocol was approved by the research ethics committee at Sohag Faculty of Medicine (Institutional Review Board Registration Number: Soh-Med-24-07-03PD). Written consents from the parents/legal guardians of the participants were obtained. Confidentiality of the participants' data was ensured.

### 2.6. Statistical Analysis

Statistical analysis was performed using IBM SPSS Statistics for Windows, version 22.0 (IBM Corp., Armonk, NY, USA). The Kolmogorov–Smirnov test was used to assess the normality of continuous variables' distributions. Normally distributed continuous variables were presented as mean ± standard deviations (SDs), while nonnormally distributed variables were presented as median (interquartile range [IQR]). Categorical variables were expressed as numbers and percentages.

Independent sample *t*-test was used to compare continuous variables with normal distributions between children and adolescents with T1DM and celiac disease and their matched controls. The Mann–Whitney *U* test was used to compare continuous variables with nonparametric distributions between both groups. The Chi-square test was used to compare categorical variables between cases and controls. A *p*-value of 0.05 or less was considered statistically significant.

## 3. Results

The study included 47 T1DM children and adolescents who had a biopsy-proven diagnosis of celiac disease between January 2017 and December 2021 and 94 age-, sex-, and duration of diabetes-matched control children and adolescents with T1DM who had persistently negative celiac screening tests during the same period.

The age, sex, and duration of T1DM of the study participants are shown in Table 1. Twenty-three children and adolescents (48.9%) with celiac disease had villous atrophy (Marsh stage 3) in the diagnostic small bowel biopsies.

Table 2 shows the insulin doses and HbA1c levels for T1DM children and adolescents with and without celiac disease. Children and adolescents with celiac disease had significantly lower TDD at diagnosis of celiac disease (*p*=0.002). However, they had a significant increase in their TDD over the following year (*p*=0.04). There were no significant differences between both groups regarding the HbA1c level at diagnosis (*p*=0.27) or after 1 year (*p*=0.81).

Table 3 shows fasting lipid profiles for T1DM children and adolescents at screening or diagnosis of celiac disease and after 1 year. There were no significant differences between children and adolescents with and without celiac disease regarding the fasting lipid profiles at screening or diagnosis of celiac disease or after 1 year.

Table 4 shows the growth parameters for T1DM children and adolescents with and without celiac disease. Children and adolescents with celiac disease had significantly lower weight, height and BMI SDS at diagnosis of celiac disease and after 1 year compared to children and adolescents without celiac disease. However, there were no significant differences in the rate of change of SDS of these growth parameters in both groups over the following year.

Table 5 shows glycemic control and growth parameters for T1DM children and adolescents with celiac disease according to the presence or absence of villous atrophy in diagnostic small bowel biopsies compared to their matched controls.

Children and adolescents with villous atrophy (Marsh stage 3) at diagnostic small bowel biopsies had significantly lower TDD at diagnosis (*p*=0.01) and significantly higher HbA1c levels after 1 year (*p*=0.04) compared to their matched controls. Similarly, children and adolescents without villous atrophy at diagnostic biopsies had significantly lower TDD at diagnosis of celiac disease. However, there were no significant differences in TDD or HbA1c levels after 1 year between children and adolescents without villous atrophy and their matched controls. Moreover, there were no significant differences in fasting serum lipid profiles at diagnosis or after 1 year between children and adolescents with and without villous atrophy and their matched controls.

Weight, height, and BMI SDS were significantly lower in children and adolescents with villous atrophy both at diagnosis of celiac disease and after 1 year compared to their matched controls. On the contrary, there were no significant differences between children and adolescents without villous atrophy and their matched controls regarding these growth parameters at diagnosis of celiac disease or after 1 year.

Table 6 shows glycemic control and growth parameters for T1DM children and adolescents with celiac disease according to the celiac autoantibody status 1 year after diagnosis compared to their matched controls.

Twenty-eight (59.6%) children and adolescents with celiac disease had normalized celiac autoantibody titers 1 year after diagnosis of celiac disease. Children and adolescents with normalized celiac autoantibodies had significantly lower TDD after 1 year compared to their matched controls (*p*=0.03). They also had lower HbA1c levels after 1 year, but this was statistically insignificant (8.55% ± 0.81% vs. 9.01% ± 1.46%; *p*=0.07). There were no significant differences in the rate of changes of weight, height and BMI SDS after 1 year between children and adolescents with normalized celiac autoantibodies and those with persistently positive celiac autoantibodies and their matched controls.

## 4. Discussion

Both celiac disease and its management with GFD might affect the metabolic control and growth parameters among children and adolescents with T1DM [42]. Dietary gluten induces an immune response that causes intestinal mucosal damage and subsequent malabsorption of essential nutrients [43]. Adherence to GFD and elimination of gluten from the diet help to restore normal intestinal mucosa and improve absorption of these nutrients. However, the rate of intestinal mucosal healing varies and is affected by the degree of adherence to GFD [44].

The current study found that T1DM children and adolescents with celiac disease had significantly lower TDDs at the diagnosis of celiac disease. The rate of increase of the TDDs throughout the following year was significantly higher among those children and adolescents compared to their matched controls. Moreover, the study demonstrated that T1DM children and adolescents with villous atrophy at diagnostic small bowel biopsies had significantly higher HbA1c levels after 1 year.

Similarly, Saadah [13] found that children with T1DM had lower daily insulin doses at diagnosis of celiac disease, with a significant increase in their insulin requirements over the following 12 months. Abid et al. [17] demonstrated that the daily insulin doses increased significantly after the diagnosis of celiac disease. Moreover, Sun et al. [15] reported that children and adolescents with T1DM had lower HbA1c levels at diagnosis of celiac disease, with a significant increase in their HbA1c levels during the first year after diagnosis of celiac disease. However, several other studies reported no significant changes in insulin doses or HbA1c levels before and after diagnosis of celiac disease [17, 28, 45, 46].

Lower insulin requirements at diagnosis of celiac disease might be attributed to malabsorption of nutrients, including glucose, secondary to intestinal mucosal damage [47]. The recovery of the intestinal mucosa on GFD might increase intestinal glucose absorption, resulting in higher postprandial blood glucose levels and worsening glycemic control [46, 48]. In addition, gluten-free foods have higher glycemic indices compared to gluten-containing foods. This induces faster and higher postprandial blood glucose excursions with worsening glycemic control in T1DM children and adolescents on GFD [48, 49].

The present study found that weight, height, and BMI SDS were significantly lower in children and adolescents with villous atrophy both at diagnosis of celiac disease and after 1 year compared to their matched controls. On the contrary, there were no significant differences between children and adolescents without villous atrophy and their matched controls regarding these growth parameters at diagnosis of celiac disease or after 1 year.

Several previous studies reported that there were no significant changes in the growth parameters for children and adolescents with T1DM following diagnosis and treatment of celiac disease [15, 17, 28]. However, Saadah [13] found that weight and BMI SDS significantly increased after diagnosis and treatment of celiac disease. Akirov et al. [18] reported that strict adherence to a GFD was associated with a significant increase in the height SDS among children and adolescents with T1DM 2 years after diagnosis of celiac disease. Moreover, Nagl et al. [27] demonstrated that weight and height SDS improved in T1DM children and adolescents with celiac disease who maintained negative celiac autoantibodies for 6 years after diagnosis of celiac disease.

The discrepancies in the findings of these previous studies could be attributed to several factors. First, the severity of clinical and histological manifestations of celiac disease might impair the growth parameters of children and adolescents at diagnosis of celiac disease [50]. Second, the degree of adherence to GFD among children and adolescents with celiac disease could determine the rate of intestinal mucosal healing and consequently the changes in their metabolic control and growth parameters [51]. Finally, the duration of follow-up for children and adolescents on GFD might affect the results of these studies, as healing of the intestinal mucosa might take years [52].

In addition, the nutritional adequacy of GFD might affect the rate of improvement in growth parameters. Exclusion of cereals from the diet might result in deficiencies in vitamin B complex, vitamin D, folic acid, and dietary fiber. Moreover, calcium, magnesium, iron, and zinc deficiencies might be associated with GFD [53]. These nutritional deficiencies might prevent the improvements in growth parameters following the use of GFD [54].

The present study demonstrated that 28 (59.6%) children and adolescents with celiac disease had normalized celiac autoantibody titers 1 year after diagnosis of celiac disease. Normalization of celiac autoantibody titer might be considered an indirect marker for adherence to GFD [38]. Previous studies have shown that celiac autoantibodies were useful in monitoring compliance with the GFD qualitatively and quantitatively [55, 56]. However, the celiac disease autoantibody titers usually return to the normal range within several months to 2 years following a strict GFD [57, 58]. Studies from different countries reported that about 25% to 75% of children and adolescents with celiac disease did not strictly follow the GFD [59, 60]. The nonadherence rate is much higher among children and adolescents with T1DM and celiac disease [23].

The present study found that children and adolescents with normalized celiac autoantibodies had significantly lower TDDs after 1 year compared to their matched controls. This might reflect better adherence to dietary instructions regarding carbohydrate intake among T1DM children and adolescents strictly adherent to GFD.

Moreover, the current study found no significant differences in the rate of changes in weight, height, and BMI SDS after 1 year between children and adolescents with normalized celiac autoantibodies and those with persistently positive celiac autoantibodies and their matched controls. Similarly, Radlovic et al. [61] did not find significant differences in the height and weight SDS among children with celiac disease who were adherent to GFD compared to nonadherent children. These findings might indicate that the rate of mucosal healing is independent of the celiac autoantibody status [62].

The present study did not find significant differences in fasting serum TC, TG, LDL-cholesterol, and HDL-cholesterol among children and adolescents with celiac disease and their matched controls at the time of diagnosis or after 1 year of treatment. A few previous studies assessed the effects of celiac disease and GFD on lipid profiles among children and adolescents with T1DM. Jessup et al. [63] demonstrated that children with T1DM had lower HDL-cholesterol levels at diagnosis of celiac disease. Other studies reported that serum HDL-cholesterol [25], TC and TG levels [26] improved on GFD.

The current study had some limitations. First, it was a single-center study. Larger multicenter studies are required to confirm the findings of this study. Second, screening for celiac disease in children and adolescents with T1DM at our unit was not done at the onset of T1DM. This delay in screening and diagnosis of celiac disease might have negatively impacted their growth parameters. Moreover, delayed diagnosis of celiac disease could result in delayed puberty of the affected children with further impairment of their growth outcomes. More studies are needed to assess the effect of celiac disease diagnosis at the onset of T1DM on pubertal timing and growth parameters. Third, the study assessed the glycemic control and growth parameters in children and adolescents with T1DM for just 1 year after diagnosis of celiac disease. Long-term follow-up studies are required to evaluate these effects after healing of the intestinal mucosa.

## 5. Conclusions

Children and adolescents with T1DM and celiac disease had lower insulin requirements and growth parameters at the diagnosis of celiac disease. The presence of villous atrophy at diagnostic small bowel biopsy was associated with worsening glycemic control after 1 year. No significant improvements in growth parameters were found 1 year after diagnosis of celiac disease. Longer follow-up periods might be required to detect improvements in growth parameters in children and adolescents with celiac disease on GFD.

## Figures and Tables

**Table 1:** Baseline characteristics of the study participants.

Variables	Children and adolescents with celiac disease (*n* = 47)	Children and adolescents without celiac disease (*n* = 94)
Age (years), median (IQR)	7.5 (5.0–10.0)	7.5 (5.0–10.1)
Sex		
Male, *n* (%)	21 (44.7%)	42 (44.7%)
Female, *n* (%)	26 (55.3%)	52 (55.3%)
Duration of T1DM (years), median (IQR)	2 (1.5–4.8)	2 (1.5–4.3)
Pathological changes in diagnostic small bowel biopsy		
Marsh Stage 1, *n* (%)	8 (17.0%)	Not applied
Marsh Stage 2, *n* (%)	16 (34.0%)	
Marsh Stage 3, *n* (%)	23 (48.9%)	
Marsh 3a, *n* (%)	12 (25.5%)	
Marsh 3b, *n* (%)	6 (12.8%)	
Marsh 3c, *n* (%)	5 (10.6%)	

Abbreviations: IQR, interquartile range; T1DM, type 1 diabetes mellitus.

**Table 2:** Insulin doses and glycated hemoglobin levels for children and adolescents with T1DM at screening or diagnosis of celiac disease and after 1 year.

Variables	Children and adolescents with celiac disease (*n* = 47)	Children and adolescents without celiac disease (*n* = 94)	*p*-Value
TDD (U/kg/day) at diagnosis, median (IQR)	0.83 (0.71–0.92)	0.90 (0.84–0.93)	0.002
TDD (U/kg/day) after 1 year, median (IQR)	0.88 (0.79–0.93)	0.91 (0.86–0.94)	0.06
Change in TDD (U/kg/day) after 1 year, median (IQR)	0.03 (−0.01 to 0.08)	0.01 (−0.01 to 0.03)	0.04
HbA1c (%) at diagnosis, mean ± SD	8.74 ± 0.98	8.97 ± 1.41	0.27
HbA1c (%) after 1 year, mean ± SD	8.89 ± 0.97	8.95 ± 1.39	0.81
Change in HbA1c (%) after 1 year, mean ± SD	0.15 ± 0.82	−0.02 ± 0.57	0.18

Abbreviations: HbA1c, glycated hemoglobin A1c; IQR, interquartile range; SD, standard deviation; T1DM, type 1 diabetes mellitus; TDD, total daily insulin dose.

**Table 3:** Fasting lipid profiles for children and adolescents with T1DM at screening or diagnosis of celiac disease and after 1 year.

Variables	Children and adolescents with celiac disease (*n* = 47)	Children and adolescents without celiac disease (*n* = 94)	*p*-Value
Serum cholesterol (mg/dL) at screening/diagnosis, median (IQR)	165 (137–209)	163 (144–185)	0.60
Serum triglycerides (mg/dL) at screening/diagnosis, median (IQR)	70 (53–82)	68 (55.8–98.5)	0.69
Serum HDL-cholesterol (mg/dL) at screening/diagnosis, median (IQR)	54 (50–59)	53 (48–63.3)	0.72
Serum LDL-cholesterol (mg/dL) at screening/diagnosis, median (IQR)	101 (80–127)	91 (78–109)	0.27
Serum cholesterol (mg/dL) after 1 year, median (IQR)	170 (140–204)	162.5 (144–186.3)	0.56
Serum triglycerides (mg/dL) after 1 year, median (IQR)	75 (62–98)	77 (62–100.5)	0.95
Serum HDL-cholesterol (mg/dL) after 1 year, median (IQR)	52 (44–60)	53 (48–61)	0.26
Serum LDL-cholesterol (mg/dL) after 1 year, median (IQR)	98 (72–133)	91.5 (77–107.8)	0.41

Abbreviations: HDL, high-density lipoprotein; IQR, interquartile range; LDL, low-density lipoprotein; T1DM, type 1 diabetes mellitus.

**Table 4:** Growth parameters for children and adolescents with T1DM at screening or diagnosis of celiac disease and after 1 year.

Variables	Children and adolescents with celiac disease (*n* = 47)	Children and adolescents without celiac disease (*n* = 94)	*p*-Value
Weight SDS at diagnosis, median (IQR)	−0.40 (−1.15 to 0.27)	0.12 (−0.59 to 0.61)	0.001
Weight SDS after 1 year, median (IQR)	−0.39 (−1.28 to 0.10)	0.24 (−0.54 to 0.69)	<0.001
Change in weight SDS after 1 year, median (IQR)	−0.01 (−0.21 to 0.31)	0.04 (−0.13 to 0.23)	0.63
Height SDS at diagnosis, mean ± SD	−1.05 ± 1.38	−0.39 ± 0.88	0.004
Height SDS after 1 year, mean ± SD	−1.14 ± 1.26	−0.42 ± 0.81	0.001
Change in height SDS after 1 year, mean ± SD	0.05 ± 0.40	0.06 ± 0.27	0.21
BMI SDS at diagnosis, median (IQR)	0.23 (−0.93 to 0.71)	0.48 (−0.12 to 0.90)	0.04
BMI SDS after 1 year, median (IQR)	0.34 (−0.53 to 0.88)	0.52 (0.03–1.08)	0.03
Change in BMI SDS after 1 year, mean ± SD	0.13 ± 0.46	0.09 ± 0.37	0.56

Abbreviations: BMI, body mass index; HbA1c, glycated hemoglobin A1c; IQR, interquartile range; SD, standard deviation; SDS, standard deviation score; T1DM, type 1 diabetes mellitus; TDD, total daily insulin dose.

**Table 5:** Glycemic control and growth parameters for T1DM children and adolescents with celiac disease according to the presence or absence of villous atrophy in diagnostic small bowel biopsy in comparison to their matched controls.

Variables	Children and adolescents with villous atrophy at diagnostic biopsy (*n* = 23)	Matched control (*n* = 46)	*p*-Value	Children and adolescents without villous atrophy at diagnostic biopsy (*n* = 24)	Matched control (*n* = 48)	*p*-Value
TDD (U/kg/day) at diagnosis, median (IQR)	0.83 (0.71–0.91)	0.89 (0.86–0.93)	0.01	0.83 (0.71–0.93)	0.90 (0.82–0.94)	0.04
TDD (U/kg/day) after 1 year, median (IQR)	0.89 (0.79–0.92)	0.91 (0.86–0.93)	0.21	0.87 (0.75–0.95)	0.89 (0.85–0.96)	0.09
HbA1c (%) at diagnosis, mean ± SD	8.81 ± 0.94	8.71 ± 1.25	0.74	8.68 ± 1.02	9.22 ± 1.52	0.08
HbA1c (%) after 1 year, mean ± SD	9.19 ± 0.79	8.69 ± 1.23	0.04	8.62 ± 1.06	9.19 ± 1.50	0.06
Weight SDS at diagnosis, median (IQR)	−0.41 (−1.15 to −0.11)	0.39 (−0.52 to 0.89)	0.002	−0.40 (−1.26 to 0.39)	−0.08 (−0.72 to 0.41)	0.26
Weight SDS after 1 year, median (IQR)	−0.45 (−1.45 to 0.04)	0.41 (−0.56 to 0.90)	0.001	−0.36 (−1.26 to 0.43)	0.02 (−0.53 to 0.52)	0.13
Change in weight SDS after 1 year, median (IQR)	−0.01 (−0.21 to 0.18)	0.04 (−0.20 to 0.20)	0.62	0.04 (−0.20 to 0.42)	0.04 (−0.07 to 0.24)	0.91
Height SDS at diagnosis, mean ± SD	−1.29 ± 1.43	−0.39 ± 0.85	0.009	−0.82 ± 1.33	−0.39 ± 0.92	0.11
Height SDS after 1 year, mean ± SD	−1.31 ± 1.28	−0.39 ± 0.81	0.004	−0.98 ± 1.25	−0.44 ± 0.83	0.06
Change in Height SDS after 1 year, mean ± SD	−0.01 ± 0.34	−0.005 ± 0.19	0.91	−0.16 ± 0.27	−0.05 ± 0.24	0.11
BMI SDS at diagnosis, median (IQR)	0.40 (−0.72 to 0.71)	0.66 (0.08–1.27)	0.04	−0.02 (−1.10 to 0.78)	0.39 (−0.47 to 0.66)	0.35
BMI SDS after 1 year, median (IQR)	0.34 (−0.38 to 0.88)	0.75 (0.09–1.28)	0.03	0.28 (−0.85 to 0.87)	0.47 (−0.04 to 0.81)	0.39
Change in BMI SDS after 1 year, mean ± SD	0.04 ± 0.54	0.03 ± 0.34	0.98	0.22 ± 0.36	0.14 ± 0.39	0.4

Abbreviations: BMI, body mass index; HbA1c, glycated hemoglobin A1c; IQR, interquartile range; SD, standard deviation; SDS, standard deviation score; T1DM, type 1 diabetes mellitus; TDD, total daily insulin dose.

**Table 6:** Glycemic control and growth parameters for T1DM children and adolescents with celiac disease according to the celiac autoantibodies status after 1 year in comparison to their matched controls.

Variables	Children and adolescents with normalized celiac autoantibodies after 1 year (*n* = 28)	Matched control (*n* = 56)	*p*-Value	Children and adolescents with persistently positive celiac autoantibodies after 1 year (*n* = 19)	Matched control (*n* = 38)	*p*-Value
TDD (U/kg/day) at diagnosis, median (IQR)	0.84 (0.71–0.92)	0.90 (0.83–0.93)	0.01	0.82 (0.75–0.92)	0.89 (0.86–0.93)	0.08
TDD (U/kg/day) after 1 year, median (IQR)	0.87 (0.71–0.92)	0.91 (0.85–0.94)	0.03	0.89 (0.81–0.95)	0.90 (0.86–0.93)	0.69
HbA1c (%) at diagnosis, mean ± SD	8.61 ± 0.93	9.09 ± 1.50	0.08	8.94 ± 1.04	8.79 ± 1.27	0.66
HbA1c (%) after 1 year, mean ± SD	8.55 ± 0.81	9.01 ± 1.46	0.07	9.41 ± 0.98	8.85 ± 1.29	0.1
Weight SDS at diagnosis, median (IQR)	−0.51 (−1.32 to 0.28)	−0.02 (−0.79 to 0.53)	0.01	−0.33 (−0.90 to 0.10)	0.23 (−0.31 to 0.63)	0.03
Weight SDS after 1 year, median (IQR)	−0.53 (−1.4 to 0.29)	0.14 (−0.63 to 0.74)	0.009	−0.33 (−1.21 to 0.04)	0.26 (−0.43 to 0.69)	0.02
Change in Weight SDS after 1 year, median (IQR)	−0.005 (−0.19 to 0.27)	0.07 (−0.15 to 0.23)	0.63	−0.01 (−0.24 to 0.41)	0.005 (−0.12 to 0.21)	0.84
Height SDS at diagnosis, mean ± SD	−1.19 ± 1.43	−0.35 ± 0.97	0.008	−0.84 ± 1.32	−0.44 ± 0.73	0.23
Height SDS after 1 year, mean ± SD	−1.26 ± 1.31	−0.39 ± 0.91	0.003	−0.96 ± 1.20	−0.45 ± 0.67	0.10
Change in height SDS after 1 year, mean ± SD	−0.06 ± 0.35	−0.04 ± 0.20	0.78	−0.12 ± 0.24	−0.01 ± 0.24	0.11
BMI SDS at diagnosis, median (IQR)	0.24 (−1.15 to 0.69)	0.39 (−0.29 to 0.84)	0.23	0.23 (−0.72 to 1.06)	0.56 (0.15–1.21)	0.08
BMI SDS after 1 year, median (IQR)	0.33 (−0.78 to 0.76)	0.52 (−0.11 to 0.88)	0.16	0.34 (−0.53 to 0.95)	0.52 (0.17–1.14)	0.13
Change in BMI SDS after 1 year, mean ± SD	0.14 ± 0.52	0.13 ± 0.37	0.94	0.12 ± 0.38	0.03 ± 0.37	0.37

Abbreviations: BMI, body mass index; HbA1c, glycated hemoglobin A1c; IQR, interquartile range; SD, standard deviation; SDS, standard deviation score; T1DM, type 1 diabetes mellitus; TDD, total daily insulin dose.

## Data Availability

The data that support the findings of this study are available from the corresponding author upon reasonable request.
